# Isolation and Characterization of Kimchi Starters *Leuconostoc mesenteroides* PBio03 and *Leuconostoc mesenteroides* PBio104 for Manufacture of Commercial Kimchi

**DOI:** 10.4014/jmb.2001.01011

**Published:** 2020-04-09

**Authors:** Kang Wook Lee, Geun Su Kim, A Hyong Baek, Hyun Sun Hwang, Do Young Kwon, Sang Gu Kim, Sang Yun Lee

**Affiliations:** Pulmuone Institute of Technology, Cheongju 28164, Republic of Korea

**Keywords:** Kimchi fermentation, lactic acid bacteria, kimchi starter, *Leuconostoc mesenteroides*, *mannitol production*

## Abstract

This study was focused on developing and obtaining a kimchi starter for use in commercial kimchi production. Kimchi varieties made with selected starters are of high quality, have high levels of mannitol, and extended shelf life. The starters were screened for properties such as mannitol production, low gas/acid production, and acid resistance. Finally, kimchi fermentation testing was performed using selected LAB starters. Kimchi samples were prepared with lactic acid bacteria (LAB) starters, including *Leuconostoc mesenteroides* PBio03 and *Leuconostoc mesenteroides* PBio104. The LAB starters are isolated from kimchi and can grow under pH 3.0 and low temperature conditions of 5°C. Four kimchi samples were fermented and stored for 28 days at 5°C. The kimchi samples made with starters (PBio03 and PBio104) had better quality (production of mannitol and maintenance of heterofermentative LAB dominance) than the non-starter kimchi samples. In the starter kimchi, *Leu. mesenteroides* was the dominant LAB, comprising 80% and 70% of total LAB counts at 7 and 21 days, respectively. Mannitol content of the kimchi with *Leu. mesenteroides* PBio03 was 1,423 ± 19.1 mg/ 100 g at 28 days, which was higher than that of the non-starter kimchi sample (1,027 ± 12.2 mg/100 g). These results show the possibility of producing kimchi with improved qualities using *Leu. mesenteroides* PBio03 and PBio104 as starters.

## Introduction

Kimchi is a traditional Korean food consisting of vegetables such as Chinese cabbage, radish, and various other ingredients including red pepper, garlic and ginger which are fermented by lactic acid bacteria (LAB) under low temperature conditions (approximately 0–10°C) to ensure optimal ripening and preservation [1]. The production of organic acids from carbohydrates during kimchi fermentation and the resulting reductions in pH maintain the freshness of kimchi during storage [2, 3]. Natural kimchi fermentation leads to the growth of various LAB, causing variations in the quality. The use of LAB starters has been considered as an alternative method for industrial production of standardized kimchi, and the purposes of starters to kimchi include sensory characteristic improvement, shelf life extension, and induction of functional properties.

Many studies on kimchi microbial communities have been performed using culture-dependent and - independent approaches. These studies have shown that LAB, including *Leuconostoc* spp., *Lactobacillus* spp., and *Weissella* spp., play the most important roles in kimchi fermentation [4]. In natural kimchi fermentation, heterofermentative LAB such as *Leuconostoc* spp. and *Weissella* spp. predominate in the early and middle stages of fermentation [2]. As the kimchi changes and becomes more acidic, homofermentative LAB such as *Lactobacillus* spp. become predominant. This predominance can lead to kimchi being excessively acidic in taste and having a soft texture [5]. Yeasts in kimchi fermentation are responsible for softening the texture of kimchi as well as generating off-flavors during the over-ripening stage, thereby producing a product of unsuitable quality [6, 7]. It takes almost 1 month to reach the over-ripening stage from the initiation of kimchi fermentation. Therefore, kimchi manufacturers have assigned a shelf life of 28 days to industrial kimchi even under refrigerated storage conditions [2].

The non-starter fermentation process primarily occurs by microorganisms naturally present in the raw materials. Such a natural and randomly occurring fermentation process depends on several conditions and cannot be controlled; thus, the product quality can unpredictably vary from batch to batch [8]. However, in starter fermentation, the changes in microorganisms indicate a different pattern.

Generally, mannitol is produced via catalytic hydrogenation of fructose and sucrose. Among LAB, only heterofermentative species are known to convert fructose into mannitol, and species belonging to the genera *Leuconostoc*, *Oenococcus,* and *Lactobacillus* have been reported to effectively produce mannitol [9-11].

Here, we screened and selected a kimchi starter, namely *Leuconostoc* spp., for mannitol production, low gas/acid production, and strong acid resistance. Kimchi samples were prepared using kimchi starter culture of *Leuconostoc mesenteroides* PBio03 and *Leu. mesenteroides* PBio104. The characteristic properties of kimchi samples were tested over a period of 28 days at 5°C.

## Materials and Methods

### Isolation, Screening and Identification of LAB (Kimchi Starter)

Approximately 250 strains were isolated from kimchi samples purchased at local markets in Daegu and Jinju, Republic of Korea, in the spring of 2019. Each of the samples was diluted with sterilized 0.85% (w/v) NaCl solution via ten-fold serial dilution. Next, 0.1 ml from each of the diluted samples was serially diluted with 9 ml saline (3M Diluent). These subsequently diluted samples were plated onto De Man Rogosa Sharpe (MRS; Becton, Dickinson and Company, USA) agar to screen for LAB strains. The plates were incubated at 30°C for 24 h. After incubation, the colonies were examined and a single colony was selected.

The isolated strains were inoculated into 10 ml of in vitro MRS broth contained in a Durham tube measuring 30 mm in height and subsequently cultured at 25°C for 24 h. The height of gas collected in the Durham tube was measured to determine the degree of gas generation, and strains that generated gas up to ≤5 mm were selected. The isolated strain colonies were each inoculated into 10 ml of MRS broth. For measuring acid production, pH was measured using a pH meter after incubation at 25°C for 24 h, and strains having a pH of ≥4.4 were selected. The tests for acid tolerance of strains to pH 2, 3 and 4 were performed following the methods by Lee *et al.* [12]. The last screened properties of kimchi starter were production of mannitol. The growth of starter strains was measured in MRS broth at different conditions such as temperature (5°C, 10°C, 15°C, 20°C, 30°C, and 40°C), pH (pH 2.0, pH 3.0, and pH 4.0) and NaCl concentrations (3%, 5%, and 7%).

Screened strains were identified by 16S rRNA gene sequencing which was amplified by PCR. PCR was performed using the primers 27F (5′-AGAGTTTGATCMTGGCTCAG-3′) and 1492R (5′-TACGGYTACCTT GTTACGACTT-3′). PCR conditions were 35 cycles at 95°C for 1 min, 55°C for 1 min, and 72°C for 2 min. PCR- amplified products (approximately 1400 base pairs) were confirmed by performing agarose gel electrophoresis, and nucleotide sequences were determined at Macrogen Co. (https://dna.macrogen.com/kor/index.jsp). The BLAST program was used to identify homologous 16S rRNA gene sequences in the database (http://www.ncbinlm.nih.gov/BLAST ).

### Preparation of Kimchi Samples

Kimchi was prepared using Chinese cabbage as the main raw material. Chinese cabbage was soaked in 9–13% (w/v) salt solution for 24 h. The soaked Chinese cabbage was washed with tap water three times, and the excess water was drained off for 3 h. Kimchi seasoning was prepared by mixing shredded radish, red pepper, garlic, ginger, onion, green onion, jeotgal, and sucrose at a ratio of 55: 9.5: 7.5: 1: 7.5: 7.5: 9.5: 2.5. The seasoning was added to the salted Chinese cabbage at a ratio (w/w) of 70:30 (Chinese cabbage: kimchi seasoning). The starter strains were grown in MRS broth overnight at 30°C, and cells were recovered via centrifugation. The cells were washed two times with sterile water and resuspended in 2 ml sterile water. The cells were added to the Chinese cabbage together with the seasoning. In this study, *Leu. mesenteroides* PBio03 and PBio104 were the starters used for kimchi samples C and D, respectively. The starter culture used for kimchi sample B is the same as that used in kimchi fermentation at present, *i.e*., the culture was a mixture of three kinds of LAB (*Leu. mesenteroides*, *Leu. citreum*, and *Lactobacillus plantarum*). The prepared kimchi samples were placed in plastic containers, each weighing 1 kg, and stored at 5°C for 28 days. The kimchi samples were examined weekly.

### pH, Total Acidity, and Mannitol Analysis of Kimchi Samples

Kimchi samples were macerated using a blender (Shinil electric mixer SMX-JC15MR, Korea). Then, the macerated samples were centrifuged (MicrocentrifugeABOGENE, Korea) for 20 min at 12,000 g, and the supernatants were tested for pH and total acidity (TA). The pH was measured using a pH meter (Orion STAR A211 pH meter, Thermo Scientific, USA), while the filtrates were titrated with 0.1 N NaOH to a pH of 8.3 for TA analysis. The supernatant samples of kimchi were analyzed for the presence of mannitol using high-performance liquid chromatography (HPLC, 1100 series, Agilent Co., USA) employing an Asahipak NH2P-50 4E (Column size 4.6 mm I.D. × 250 mm, Shodex, Japan), and an RI detector (1260 series, Agilent Co.). An acetonitrile and water mixture (75: 25, v/v) was used as the mobile phase at 1.5 ml/min.

### Microbial Analysis of Kimchi Samples

Kimchi samples were mixed with 0.85% NaCl solution and homogenized for 2 min using a stomacher (BagMixer 400, Interscience, France). The homogenates were serially diluted with 9 ml saline (3M Diluent, USA), and the diluted samples were spread onto a plate count agar (PCA, USA) for obtaining total viable bacteria counts as well as on Petrifilm_EC (3M Health Care, USA) for obtaining the coliform bacteria count, MRS (PCA) agar for LAB count, and Dichloran Rose Bengal Chloramphenicol (DRBC, USA) agar for yeast.

The MRS agar plates were counted after 48 h of incubation at 30°C. Thirty colonies were selected from each MRS agar plate and prepared for DNA sequencing. LAB strains were identified by 16S rRNA gene sequencing which was amplified by PCR. PCR method was mentioned previously (Isolation, Screening and Identification of LAB).

### Sensory Evaluation of Kimchi Samples

Sensory evalutions of kimchi samples were done at 7 days of storage time. The test group consisted of 5 people (men:women = 2:3) and the average age was 35.3 years. A 5-point scale for taste, color, flavor, texture and overall acceptability was used.

## Results and Discussion

### Isolation and Screening of LAB Starters

About 250 LAB strains were isolated from various kimchi samples. Screening revealed twenty-five of them to be low gas-producing and low acid-producing strains (Table 1). In addition, seven strains were found to be resistant to low pH conditions (Table 2), and when exposed to simulated in vitro gastric juice of pH 3, the survival rates ranged from 64.8–78.8%. Gas and acid production of kimchi starter are important properties in the kimchi industry, as they prevent kimchi packaging expansion and extend intake period, respectively. The acid resistance affects the dominance rate of kimchi starter during fermentation. Finally, screening was carried out to select a starter strain for high production of mannitol. The results of the mannitol production of tested strains in MRS are presented in Table 3. In MRS medium (3% fructose), mannitol concentrations of 25.0 and 19.2 g/l, were achieved by *Leu. mesenteroides* PBio03 and *Leu. mesenteroides* PBio104, respectively. Among LAB, only heterofermentative species such as *Leuconostoc* are known to convert fructose into mannitol [2, 8, 15]. In other reports, the results of mannitol production in MRS broth for *Leu. citreum* KACC 91348P, *Leu. mesenteroides* D1 and *Leu. mesenteroides* B-742C were measured at 30.4, 27.3, and 29.4 g/l (MRS broth containing 4% fructose) [11]. The strains were tested for growth in MRS broth in different conditions such as temperature, pH and NaCl concentrations. The selected strains (PBio03, PBio104) were able to grow at 5°C, pH 3.0 and 7% NaCl concentration (Table 4).

### pH and TA of Kimchi Samples

**Fig. 1:**
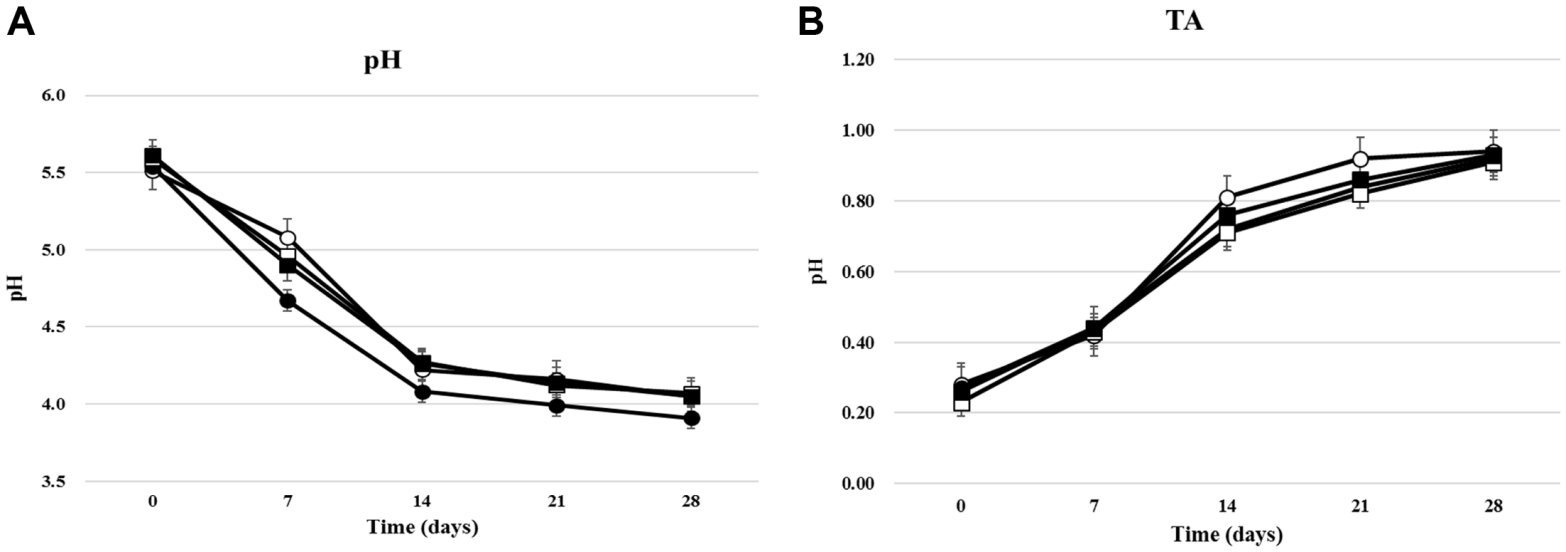
Changes of microbial populations in kimchi samples during fermentation (four weeks). (**A**) pH, (**B**) TA: ○, Kimchi A (non-starter); ●, Kimchi B (LAB mix starter); □, Kimchi C (*Leu. mesenteroides* PBio03); ■ , Kimchi D (*Leu. mesenteroides* PBio104).

The initial pH values of kimchi samples immediately after preparation were 5.51–5.61 (Fig. 1A), and all kimchi samples showed a reduction in pH during fermentation and storage (28 days). During fermentation, *Leu. mesenteroides*, a heterolactic fermentation-type LAB, and other LAB produced lactic acid and acetic acid, resulting in pH reductions in the kimchi samples. The pH of kimchi A (a non-starter sample) was 5.23 ± 0.02 at 7 days, which was the highest among kimchi samples. Other kimchi samples (LAB-added samples) showed lower pH values than kimchi A: kimchi B, 4.67; kimchi C, 4.90; and kimchi D, 4.82. The kimchi C and D samples to which the isolated LAB starter strains had been added showed higher pH values than those shown by the previously used kimchi samples containing LAB starters. The pH profile was similar to that of other reported typical kimchi fermentations [4, 13].

The TA values of the kimchi samples changed with the pH values but in a reverse direction (Fig. 1B). Kimchi samples showed TA values of 0.27–0.33. The TA values increased with fermentation time, and a significant increase occurred between 7 and 14 days, similar to the changes in pH values. This result can be attributed to the increased total LAB count between 7 and 14 days. Kimchi B showed the highest TA value of 0.82 ± 0.03 at 28 days, whereas the other kimchi samples showed TA values of 0.71–0.75. Kimchi C showed the lowest TA value (0.71 ± 0.02). Kimchi is in its best state for consumption when its TA value reaches 0.6–0.8 during fermentation [14]. Therefore, kimchi samples were well fermented and ready for consumption after 7 days, and their edibility was maintained for approximately 21 days.

### Analysis of Mannitol and Sugar Content in Kimchi Samples

**Fig. 2:**
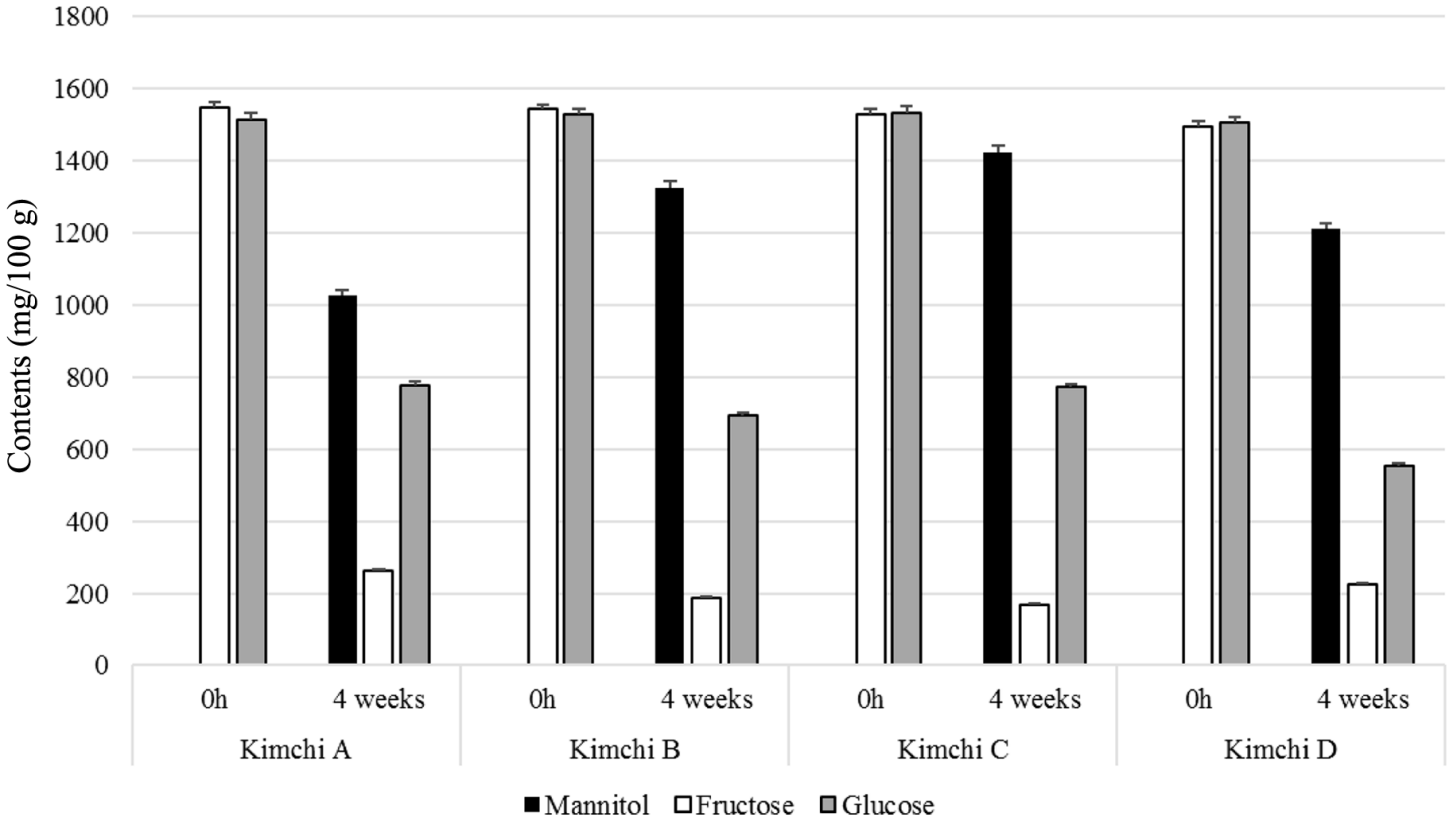
Mannitol and sugar contents of kimchi fermentation by non-starter or starter strains at 4-week storage times. Kimchi A, non-starter kimchi; Kimchi B, LAB mix starter (*Leu. mesenteroides*, *Leu. citreum* and *Lb. plantarum*); Kimchi C, *Leu. mesenteroides* PBio03 starter; Kimchi D, *Leu. mesenteroides* PBio104); Black box, mannitol; Grey box, fructose, White box, Glucose; Sucrose, Lactose, Maltose are not detected.

Mannitol content in kimchi samples was analyzed after 28 days of storage time (Fig. 2). At 28 days, mannitol content in kimchi D (containing *Leu. mesenteroides* PBio104) was 1,212 ± 9.6 mg/100 g; kimchi C (containing *Leu. mesenteroides* PBio03), 1,423 ± 19.1 mg/100 g, which was the highest; and non-starter kimchi (sample A), 1,027 ± 12.2 mg /100 g, which was the lowest. The mannitol content in kimchi C was 138.5% and 107.2% higher than that in non-starter kimchi (A) and kimchi B (mix LAB starter), respectively.

The mannitol content in kimchi B was 1,327 ± 7.2 mg/100 g at 28 days. Major free sugars detected included glucose and fructose, which have been known to play several roles as carbon sources for LAB metabolism during kimchi fermentation. In addition, we analyzed mannitol of commercially available kimchi products (competitor products). As a result, the mannitol content in the products of other companies were 1,250 mg/100 g and 960 mg/ 100 g at 28 days, each. These results should serve as an advantage in our future plan, which is to conduct field- testing within three months and apply it to some of the products at our own company. At 0 days, the fructose and glucose contents in kimchi samples were 1,497-1,548 mg/100 g and 1,505-1,534 mg/100 g, respectively (Fig. 2). However, the fructose and glucose were used for the growth of lactic acid bacteria and mannitol production, and gradually decreased. At 28 days, the fructose and glucose contents in kimchi samples decreased to 170-264 mg/ 100 g and 555-778 mg/100 g, respectively. Another study reported that mannitol production was inversely correlated with a decrease in fructose and glucose content [15].

### Microbial Analysis and Sensory Evaluation of Kimchi Samples

**Fig. 3:**
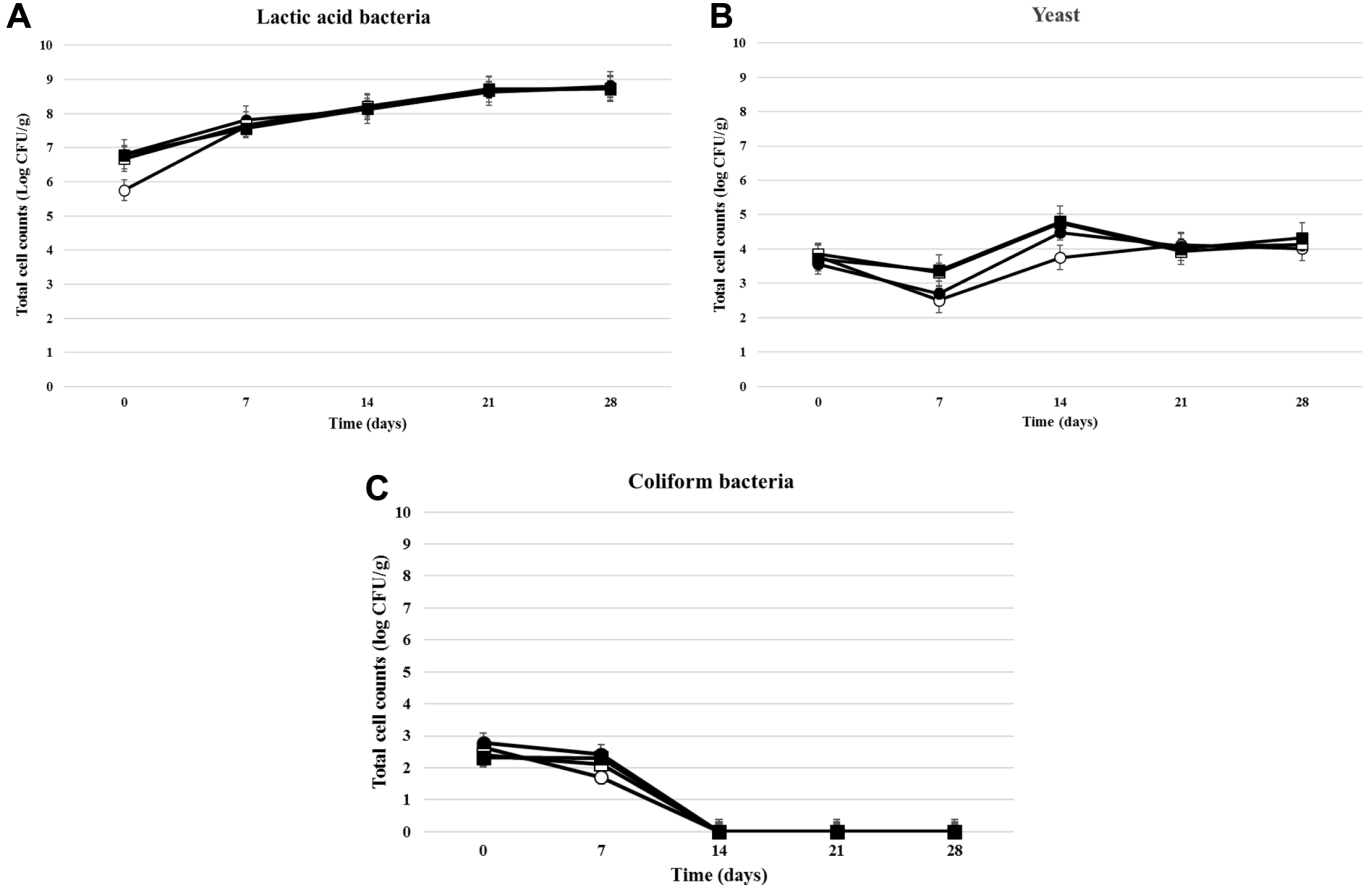
Changes of microbial populations in kimchi samples during fermentation (four weeks). (**A**) Lactic acid bacteria, (**B**) Yeast, (**C**) Coliform bacteria: ○, Kimchi A (non-starter); ●, Kimchi B (LAB mix stater); □, Kimchi C (*Leu. mesenteroides* PBio03); ■, Kimchi D (*Leu. mesenteroides* PBio104).

Immediately after the preparation of kimchi samples, the number of LAB was 5.70 × 10^5^ CFU/g for kimchi A (non-starter), and the number increased continuously to 5.90 × 10^8^ CFU/g during 28 days of storage at 5°C (Fig. 3). The initial LAB counts for kimchi C (PBio03) and kimchi D (PBio104) were 4.80 × 10^6^ CFU/g and 6.00 × 10^6^ CFU/g, which were 10-fold higher than LAB counts for kimchi A. The highest LAB counts (5.39 × 10^9^ CFU/g and 5.29 × 10^9^ CFU/g) were observed at 28 days. The changes in LAB counts from starter kimchi samples showed a similar tendency [2].

PCA agar plates were used to count the total viable bacteria in the kimchi samples, and the numbers were quite similar to the LAB counts on MRS agar plates (results not shown). The results indicate that LAB were the most abundant microorganisms associated with kimchi fermentation. Yeasts were detected from all kimchi samples. The initial yeast counts of kimchi C and D were 7.20 × 10^3^ CFU/g and 7.20 × 10^3^ CFU/g, respectively. After 7 days, the numbers decreased to 2.10–2.30 × 10^3^ CFU/g, and the counts of yeast increased to 5.75–6.24 × 10^4^ CFU/g at 14 days, after which the number gradually decreased to 1.38–2.08 × 10^4^ CFU/g at 28 days. At 7 days, kimchi C and D samples showed higher yeast counts than other kimchi samples. Yeasts appear during the later stages of kimchi fermentation and are considered undesirable for kimchi fermentation because they generate off-flavors and deteriorate the texture of kimchi by secreting pectinases [7, 16].

The initial counts of coliform bacteria were 2.10–6.00 × 10^2^ CFU/g, which gradually decreased with a decrease in pH during kimchi sample fermentation.

LAB species were identified by culture-independent methods using MRS agar plates. Thirty colonies on MRS agar plates were randomly selected from each sample (Table 5). For kimchi A (non-starter) at 7 days, 17 colonies (56.7%) had sequences identical to those of heterofermentative LAB, such as *Leu. mesenteroides* and *Weissella koreensis*. At 28 days, 8 colonies (26.7%) of the same sample (kimchi A) were sequenced and identified to match with the sequence of *Leu. mesenteroides*. Among the 30 colonies isolated from kimchi B (LAB mix starter), 13 colonies were of *Leu. mesenteroides* (43.3%), 7 colonies were of *Leu. citreum* (23.3%), and 10 colonies were of *Lb. sakei* (33.3%) at 7 days. At 28 days, 13 colonies (43.3%) of kimchi B had sequences identical to those of heterofermentative LAB. Among the 30 colonies isolated from kimchi C (PBio03), 19 colonies were of *Leu. mesenteroides* (63.3%), 8 colonies were of *Lb. sakei* (26.7%), and 2 colonies were of *Lb. plantarum* (6.7%), and 1 colony (3.3%) could not be sequenced at 28 days. Among the 30 colonies isolated from kimchi D (PBio104), 17 colonies were of *Leu. mesenteroides* (56.6%), 11 colonies were of *Lb. sakei* (36.7%), and 2 colonies were of *Lb. plantarum* (6.7%) at 28 days. Regarding the total cell counts of LAB during fermentation carried out at 30°C, the number of *Leuconostoc* spp. initially increased and was followed by a rapid decrease when the number of *Lactobacillus* species increased at pH 4.0–4.5. However, a delay was noted in the time periods associated with increase and decrease in the numbers of LAB (*Leuconostoc* spp. and *Lactobacillus* spp.) at 5°C [17]. Thus, *Leu. mesenteroides* is widely used as a kimchi starter because the strains are expected to exert favorable effects on kimchi [18].

Sensory evaluation was conducted with a small group of people (Table 6). Overall acceptability of kimchi B and C was better than that of kimchi A. There was no significant difference in the taste of kimchi B and C samples. Kimchi samples using starter received better scores for taste and overall acceptability. However, there was no difference in color, flavor or texture among the samples, thereby indicating that the addition of starter did not significantly affect any of these characteristics.

In the present study, we demonstrated the isolation and characterization of useful kimchi starter for commercial kimchi production. The kimchi using selected starter such as *Leu mesenteroides* PBio03 is of high quality due to high production of mannitol. The product also has extended shelf life and low gas/acid production. The starters were screened by several properties such as mannitol production and acid resistance, and kimchi fermentation tests were performed using selected LAB starter. These results show that *Leu mesenteroides* PBio03 and PBio104 are useful as starters for commercial kimchi fermentation.

## Figures and Tables

**Table 1:** Screening of kimchi starter by gas/acid production.

Strain No	Identified strain	Gas production (cm)	Acid production (pH)	Strain No	Identified strain	Gas production (cm)	Acid production (pH)
1	*Weissella koreensis*	++ (0.7) *	4.69	63	*Weissella koreensis*	++ (0.6)	4.60
3	*Leuconostoc mesenteroides*	++ (0.7)	4.50	67	*Weissella koreensis*	++ (0.8)	4.68
7	*Weissella koreensis*	+ (0.2)	4.70	69	*Weissella koreensis*	++ (0.5)	4.77
9	*Weissella koreensis*	+ (0.4)	4.74	71	*Weissella koreensis*	++ (0.8)	4.70
10	*Weissella koreensis*	+ (0.3)	4.72	72	*Weissella koreensis*	++ (0.8)	4.75
15	*Weissella koreensis*	+ (0.2)	4.75	88	*Weissella koreensis*	++ (0.8)	4.68
27	*Weissella koreensis*	++ (0.6)	4.71	93	*Weissella koreensis*	++ (0.7)	4.77
31	*Weissella koreensis*	++ (0.6)	4.75	10^3^	*Weissella koreensis*	++ (0.8)	4.80
32	*Weissella soli*	+ (0.4)	4.81	10^4^	*Leuconostoc mesenteroides*	++ (0.6)	4.48
37	*Weissella koreensis*	++ (0.7)	4.74	112	*Weissella koreensis*	++ (0.7)	4.50
38	*Weissella koreensis*	++ (0.7)	4.71	122	*Lactobacillus sakei* subsp. *sakei*	++ (0.7)	5.53
52	*Weissella koreensis*	++ (0.6)	4.77	185	*Lactobacillus sakei*	++ (0.8)	4.78

* +, 0.1 mm – 0.5 mm; ++, 0.6 mm – 1.0 mm; ++, 1.1 mm – 1.5 mm

**Table 2:** Acid resistance of isolated strains.

No	Strains	Acid resistance (survival rates, %)		

		pH2.0, 2h	pH3.0, 2h	pH4.0, 2h
3	*Leuconostoc mesenteroides*	20.6(±0.1)	72.4(±0.2)	90.0(±0.4)
7	*Weissella koreensis*	21.3(±0.2)	70.4(±0.3)	86.5(±0.2)
9	*Weissella koreensis*	23.5(±0.4)	68.2(±0.3)	84.0(±0.2)
15	*Weissella koreensis*	23.2(±0.3)	68.4(±0.7)	84.2(±0.5)
69	*Weissella koreensis*	14.8(±0.6)	64.8(±0.1)	74.4(±0.3)
104	*Leuconostoc mesenteroides*	21.4. (±0.6)	66.4(±0.2)	88.4(±0.3)
122	*Lactobacillus sakei* subsp. *sakei*	54.2(±0.2)	78.8(±0.2)	98.4(±0.4)

**Table 3:** Mannitol production of isolated strains.

No	Strain	Mannitol production (g/100g)
3	*Leuconostoc mesenteroides* PBio03	2.5
7	*Weissella koreensis* PBio07	0.48
9	*Weissella koreensis* PBio09	0.43
104	*Leuconostoc mesenteroides* PBio104	1.92
Con	*Leu. mesenteroides* WK32 + *Leu. citreum* KM20	1.62

**Table 4:** Growth characteristics of isolated strains.

Isolated strains	Temperature						pH			NaCl concentration		
		
	5℃	10℃	15℃	20℃	30℃	40℃	2.0	3.0	4.0	3%	5%	7%
*Leu. mesenteroides* PBio03	+	+	+	+	+	+	-	+	+	+	+	+
*Leu. mesenteroides* PBi104	+	+	+	+	+	+	-	+	+	+	+	+
*Weissella koreensis* PBio07	-	+	+	+	+	+	-	+	+	+	+	+
*Weissella koreensis* PBio09	+	+	+	+	+	-	-	+	+	+	+	+

+, Growth; -, No growth

**Table 5:** Microbial population of lactic acid bacteria during 28 days.

Storage times	samples	Species	Clone numbers out of 30 (%)
1 week	Kimchi A	*Leuconostoc mesenteroides*	14/30 (46.7)
		*Weissella koreensis*	3/30 (10.0)
		*Lactobacillus sakei*	11/30 (36.7)
		*Lactobacillus plantarum*	2/30 (6.6)
	Kimchi B	*Leuconostoc mesenteroides*	13/30 (43.3)
		*Leuconostoc citreum*	7/30 (23.3)
		*Lactobacillus sakei*	10/30 (33.3)
	Kimchi C	*Leuconostoc mesenteroides*	26/30 (86.7)
		*Weissella koreensis*	1/30 (3.3)
		*Lactobacillus sakei*	3/30 (10.0)
	Kimchi D	*Leuconostoc mesenteroides*	24/30 (80.0)
		*Weissella koreensis*	2/30 (6.7)
		*Lactobacillus sakei*	4/30 (13.3)
3 weeks	Kimchi A	*Leuconostoc mesenteroides*	11/30 (36.7)
		*Lactobacillus sakei*	17/30 (56.7)
		*Lactobacillus plantarum*	2/30 (6.6)
	Kimchi B	*Leuconostoc mesenteroides*	8/30 (26.7)
		*Leuconostoc citreum*	5/30 (16.7)
		*Lactobacillus sakei*	11/30 (26.7)
		*Lactobacillus plantarum*	6/30 (20.0)
	Kimchi C	*Leuconostoc mesenteroides*	22/30 (73.4)
		*Lactobacillus sakei*	7/30 (23.3)
		*Uncultured bacterium*	1/30 (3.3)
	Kimchi D	*Leuconostoc mesenteroides*	21/30 (70.0)
		*Lactobacillus sakei*	4/30 (13.3)
		*Lactobacillus plantarum*	5/30 (16.7)
4 weeks	Kimchi A	*Leuconostoc mesenteroides*	8/30 (26.7)
		*Lactobacillus sakei*	15/30 (50.0)
		*Lactobacillus plantarum*	4/30 (13.3)
		*Lactobacillus brevis*	3/30 (10.0)
	Kimchi B	*Leuconostoc mesenteroides*	9/30 (30.0)
		*Leuconostoc citreum*	4/30 (13.3)
		*Lactobacillus sakei*	14/30 (46.7)
		*Lactobacillus plantarum*	3/30 (10.0)
	Kimchi C	*Leuconostoc mesenteroides*	16/30 (53.3)
		*Lactobacillus sakei*	11/30 (26.7)
		*Lactobacillus plantarum*	2/30 (6.7)
		No reaction (PCR)	1/30 (3.3)
	Kimchi D	*Leuconostoc mesenteroides*	17/30 (56.6)
		*Lactobacillus sakei*	11/30 (36.7)
		*Lactobacillus plantarum*	2/30 (6.7)

**Table 6:** Sensory evaluation results of the kimchi samples using kimchi starter.

Samples	Taste	Color	Flavor	Texture	Overall acceptability
Kimchi A	3.00 ± 0.71	4.20 ± 0.84	3.40 ± 0.55	3.20 ± 0.84	3.00 ± 0.71
Kimchi B	3.80 ± 0.54	4.40 ± 0.55	4.00 ± 0.71	3.40 ± 0.55	3.40 ± 0.55
Kimchi C	3.60 ± 0.54	4.20 ± 0.45	3.60 ± 0.55	3.20 ± 0.84	3.40 ± 1.14
Kimchi D	3.20 ± 0.45	4.40 ± 0.55	3.80 ± 1.30	3.40 ± 1.51	3.00 ± 0.71
